# Case Report: A novel *KNCH2* variant-induced fetal heart block and the advantages of fetal genomic sequencing in prenatal long-term dexamethasone exposure

**DOI:** 10.3389/fgene.2022.1010078

**Published:** 2022-11-29

**Authors:** Huiping Huang, Siyuan Jing, Shaoying Wu, Li Wei, Qian Zhang, Yimin Hua, Yifei Li, Haiyan Yu, Kaiyu Zhou

**Affiliations:** ^1^ Key Laboratory of Birth Defects and Related Diseases of Women and Children of MOE, Department of Pediatrics, West China Second University Hospital, Sichuan University, Chengdu, China; ^2^ Department of Pediatrics, The First People’s Hospital of Shuangliu District, West China Airport Hospital of Sichuan University, Chengdu, China; ^3^ Department of Pediatrics, The Second People’s Hospital of Liangshan Yi Autonomous Prefecture, Xichang, China; ^4^ Key Laboratory of Birth Defects and Related Diseases of Women and Children of MOE, Department of Obstetrics and Gynecology, West China Second University Hospital, Sichuan University, Chengdu, China

**Keywords:** fetal bradycardia, long QT syndrome, kcnh2, prenatal management, dexamethasone exposure, case report

## Abstract

**Background:** Fetal bradycardia is a common but severe condition. In addition to autoimmune-mediated fetal heart block, several types of channelopathies induce high-degree atrioventricular block (AVB). Long QT syndrome (LQTS) is a major cause of non-autoimmune-mediated fetal heart block. Due to the limitations of prenatal diagnostic technologies, LQTS is seldom identified unless fetal genetic screening is performed. Thus, long-term prenatal dexamethasone (DEX) exposure can become a challenge for these patients. We report on a rare case of a novel *KCNH2* variant related to LQTS and associated with high-degree fetal AVB with long-term DEX exposure. This case led us to review our prenatal administration strategy for such patients.

**Case Presentation:** A fetus was identified with high-degree AVB (2:1 transduction at 28 + 2 gestational weeks). Typical tests of immune function in the pregnant woman were conducted including tests for thyroid function, rheumatic screening, autoimmune antibodies (such as anti-Ro/SSA and anti-La/SSB), and anti-nuclear antibodies (anti-ANA). Following the recommended protocol, the pregnant patient received DEX (0.75 mg/day) during pregnancy. Subsequently, the fetal AVB changed from 2:1 to prolonged AV intervals with ventricular tachycardia, which suggested a therapeutic benefit of DEX in some respects. However, a high-degree AVB with a significantly prolonged QTc interval was identified in the neonate following birth. Genetic testing revealed that a *KCNH2* c.1868C>A variant induced LQTS. The body length remained approximately -3.2 SD from the reference value after prenatal long-term DEX exposure, which indicated a developmental restriction. Additionally, the functional validation experiments were performed to demonstrate the prolonged duration of calcium transit both in depolarization and repolarization with the *KCNH2* c.1868C>A variant.

**Conclusion:** Genetic screening should be recommended in fetuses with autoimmune antibody negative high-degree AVB, especially for 2:1 transduction AVB and in fetuses with changes in fetal heart rhythm following initial DEX treatment. Genetic screening may help identify genetic variant–related channelopathies and avoid unexpected prenatal exposure of DEX and its possible long-term adverse postnatal complications.

## Introduction

Fetal heart block is a rare and threatening disease and is the most commonly observed type of fetal bradycardia. Among all fetal heart block patients, > 50% of cases are associated with maternal autoimmune diseases, and the incidence of fetal heart block in pregnancies with positive anti–SSA(Ro) and anti–SSB(La) test results is 2%–5% (Manolis et al., 2020). Fetuses with positive antibody screens represent an estimated 25% of neonatal lupus patients. Studies suggest that transplacental anti-nuclear antibodies (ANAs) may attack the cardiac conduction system of the fetus. Suryawanshi et al. (2020) used single-cell RNA-sequencing to determine the immunological activity of immune cells in human heart samples that were positive for anti-SSA/Ro and associated with congenital heart block (CHB). Researchers found increased and heterogeneous interferon responses in various cell types of the CHB heart compared with healthy controls, which contributed to subsequent fibrosis in the heart. Thus, in autoimmune antibody–positive pregnancies, dexamethasone (DEX) is typically administered to prevent the progression of or to cause the reversal of fetal heart block. However, there are also a large number of patients with fetal heart block without positive antibodies. Thus, the administration strategy for DEX treatment has always been challenging when weighing the benefits of DEX treatment on antibody-negative fetuses (Liao et al., 2021; Tang et al., 2021). According to the current therapeutic protocol, DEX is most recommended for such patients; yet the risks of long-term DEX administration is an ongoing debate.

Attempts have been made to identify potential causes of fetal heart block. Studies have reported that long QT syndrome (LQTS) may present as severe fetal bradycardia or as fetal heart block with a 2:1 transduction (Adler et al., 2020). LQTS does not require DEX treatment, and it is difficult to distinguish between LQTS with heart block and autoimmune-associated fetal heart block that require DEX treatment during pregnancy unless fetal genetic testing is performed to identify genetic variant–related arrhythmias (Martínez-Barrios et al., 2022). Moreover, cases with negative antibody screens commonly present postnatally with adverse arrhythmias including LQTS and high-degree AVB. LQTS is considered a clinical disorder with a genetic origin and is characterized by delayed repolarization of cardiomyocytes, electrocardiographic (ECG) QT prolongation, an elevated risk of syncope, and sudden cardiac death caused by polymorphic ventricular tachycardia that is known as torsades des pointes (TdP) (Tang et al., 2021). The loss of function variants in *KCNH2* are the leading cause of LQTS (Liao et al., 2021). Three encoding ion channel genes have been identified as responsible for most of the LQTS cases: *KCNQ1* (Kv7.1 channel) causing LQT1 (Adler et al., 2020), *KCNH2* (Kv11.1 channel) causing LQT2 (Martínez-Barrios et al., 2022), and *SCN5A* (Nav1.5 channel) causing LQTS (Dai et al., 2021). Thus, the early and timely ability to distinguish congenital LQTS and autoimmune-associated fetal heart block is critical to avoid unnecessary long-term prenatal DEX exposure.

Herein, we report on a rare case of a novel *KCNH2* variant-related LQTS with an associated high-degree fetal AVB. After prenatal DEX administration, only a prolonged AV interval was identified *in utero*. Thus, the fetus received long-term DEX exposure. Postnatal ECG and genetic testing revealed that the neonate had LQTS and a *de novo KCNH2* variant, respectively. This case led us to review our prenatal DEX administration strategy for patients and suggests that genetic testing should be conducted in autoimmune antibody-negative fetuses to avoid long-term DEX exposure given that DEX treatment leads to improved outcomes.

## Case presentation

### Ethical compliance

This report was approved by the ethics committee of the West China Second Hospital of Sichuan University (approval number 2014-034). Informed consent was obtained from the patient’s parents prior to performing whole-exon sequencing and for the inclusion of the patient’s clinical and imaging details in subsequent publications.

### History of illness

A 30-year-old pregnant woman was admitted to our center at 28 + 0 gestational weeks for a general fetal screening, and a fetal ultrasound demonstrated fetal bradycardia. Fetal echocardiography was then performed at 28 + 2 gestational weeks to determine if the fetus suffered from a high-degree AVB (2:1 transduction). The fetal echocardiography demonstrated a normal intracardiac structure and normal cardiac function with a 10 out of 10 cardiovascular profile score. Tests for immune activity in pregnant women were performed including thyroid function, rheumatic screening, autoimmune antibodies (such as anti-Ro/SSA and anti-La/SSB), and ANA. All aforementioned tests were negative. In addition, a test was performed for potential viral infections, including tests for adenovirus, coxsackie virus, and B-19 virus, and all were negative. Following the recommended protocol, the pregnant patient received DEX (0.75 mg/day) during pregnancy. Subsequently, the fetal AVB changed from 2:1 to a prolonged AV interval with ventricular tachycardia, which suggests a therapeutic benefit of DEX in some aspects in the pregnant woman administrated DEX (beginning at 4.5 mg/day with a dose reduction at 34 + 3 gestational weeks when the 2:1 transduction AVB changed to prolonged AV with an interval of 147 ms) until delivery at 37 + 0 gestational weeks. Following birth, the neonate was confirmed to have a significantly prolonged QTc interval with high-degree AVB (2:1 transduction). Transient ventricular tachycardia was identified using a Holter monitor. The neonate’s parents had no positive or related family history of arrhythmia, cardiomyopathy, congenital heart disease, or coronary artery disease. The ECGs assessments of the parents presented normal performance (Supplementary Figure S1).

**FIGURE 1:**
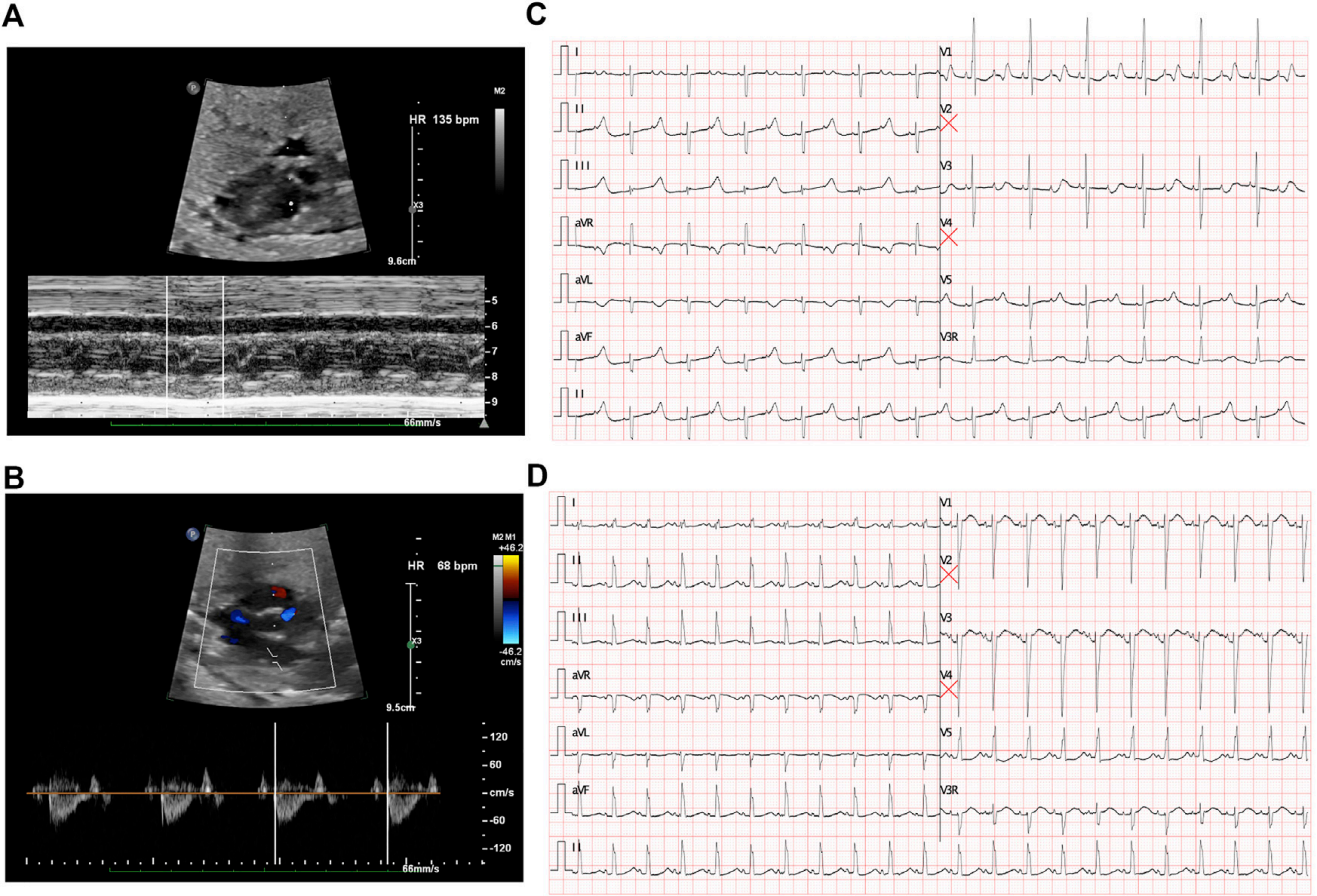
Fetal echocardiography to determine bradycardia. **(A)** M-mode echocardiography demonstrated an atrial rate of 135 beats per minute before treatment. **(B)** Doppler demonstrated a ventricular rate of 68 beats per minute before treatment and indicated a 2:1 AV block. **(C)** ECG presented a 2:1 transduction atrioventricular block postnatally. **(D)** ECG revealed a significant prolonged QTc interval after birth.

The first intrapartum fetal echocardiography exam was performed at 28 + 2 gestational weeks, and the development of the four cardiac chambers and valvar movements were normal. The atrial rate was 135 beats/min, while the ventricular rate was 68 beats/min (Figures 1A and B), and the cardiovascular system profile was normal (10/10 points). Thus, a diagnosis of fetal AVB (second degree, type II) was identified. The maternal autoimmune antibody test results were negative for anti–Ro/SSA (0.8 RU/mL, n.v. < 20 RU/mL), anti–La/SSB (<0.5 RU/mL, n.v. < 20 RU/mL), and ANAs (negative, n.v. < 1:100). The IgM result for adenovirus, coxsackie virus, and B-19 were all negative. Next, DEX (4.5 mg/day) was provided to the mother. During fetal follow-up at 34 + 3 gestational weeks, a fetal AVB of 2:1 transduction changed into a first-degree AVB with a prolonged AV interval (147 ms) as the ventricular rate was 130–142 beats/min and the cardiovascular system profile remained normal. Thus, the DEX treatment seemed to confer some benefits in this case. At this point, the dosage of DEX was reduced to 1.5 mg/day until delivery.

**FIGURE 2:**
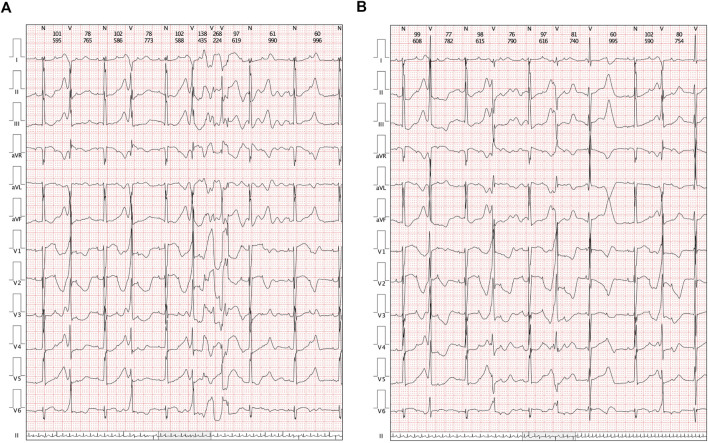
Clinical manifestations in Holters. **(A)** Transit ventricular tachycardia was identified. **(B)** Holter demonstrated a paired premature ventricular contraction.

A cesarean section was performed at 37 + 0 gestational weeks. After birth, bradycardia was also documented, and the ventricular rate was approximately 65 beats/min with an acceptable SpO_2_ of approximately 98%. An ECG of the neonate demonstrated a 2:1 transduction AVB (Figure 1C). One week later, the ECG revealed a significantly prolonged QTc interval (498–534 ms, Figure 1D). Moreover, Holter monitoring demonstrated transit ventricular tachycardia (Figure 2A) and doublet premature ventricular contractions (Figure 2B). A slight elevation of cTnI was also observed (0.162 ug/L, n.v. < 0.06 ug/L). The autoimmune antibody test results of the neonate were negative. ECG confirmed the presence of a patent foramen ovale, tricuspid regurgitation (mild) with an ejection fraction of 71%, and fractional shortening of 38%. Thus, a diagnosis of LQTS was suspected for this patient, and whole-exon sequencing was performed to identify any potential genetic variants. This was the first child between the couple.

### Molecular results

**FIGURE 3:**
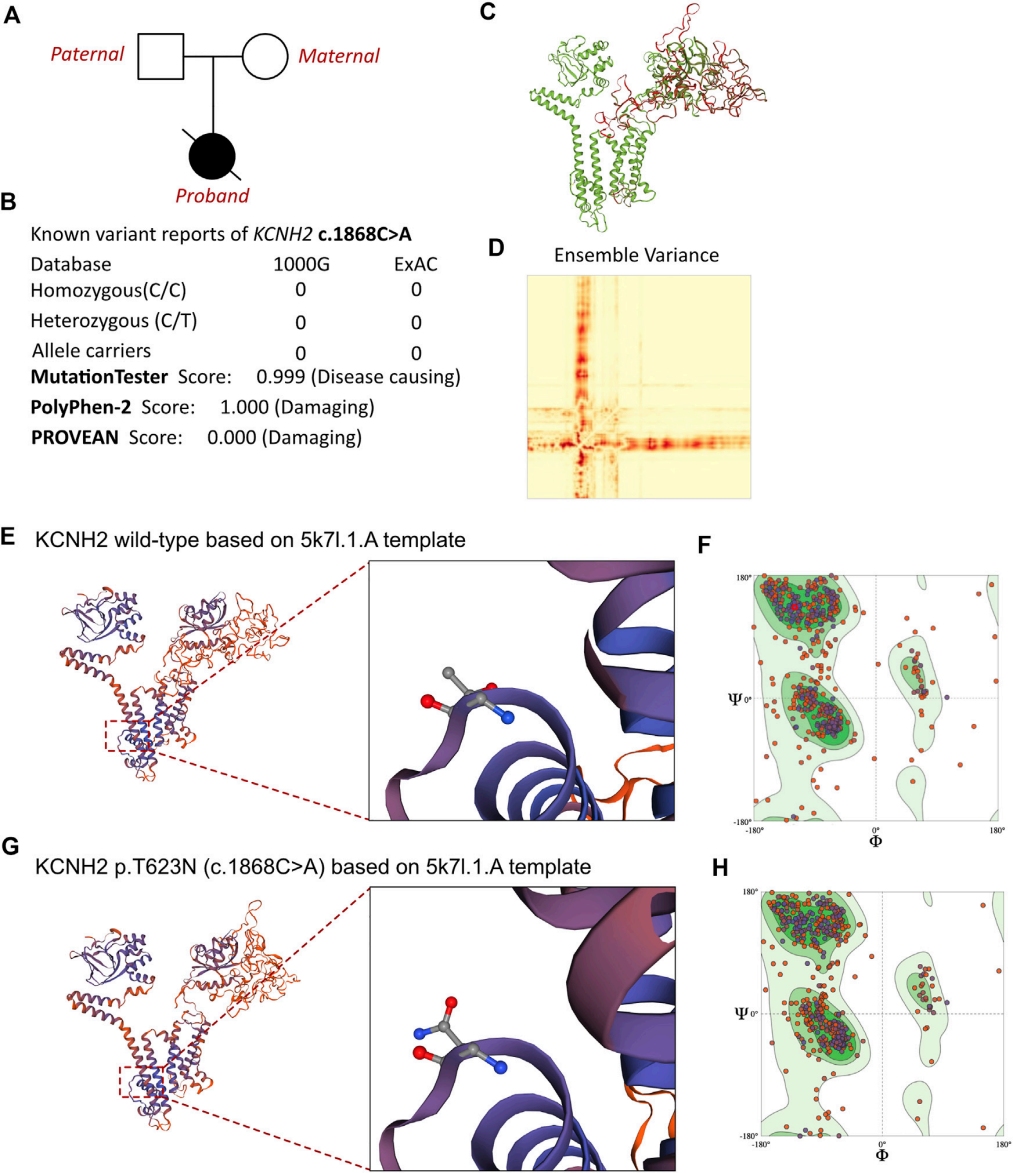
Effects of *KCNH2* c.1868C>A mutation on molecular protein structure. **(A)** Family pedigree tree of this proband. **(B)** Summary of current reports on the individuals of *KCNH2* and its predicted damages on the molecular function of the c.1868C>A variant. **(C)** SWISS-MODEL to predict the variant’s wild type and mutated protein crystal structure, and the variant impaired a large proportion of molecular structure. **(D)** Ensemble variance between the wild type and mutant protein structure. **(E,G)** SWISS-MODEL to predict the variant’s wild-type and mutated protein crystal structures using 5k7l.1.A template, and structural changes were identified. **(F)** Ramachandran plots of wild-type KCNH2. **(H)** Ramachandran plots of KCNH2 with the p.T623N variant.

To evaluate any potential genetic causes of the neonate’s condition, a peripheral blood sample was obtained from the patient in an ethylenediaminetetraacetic acid anticoagulant blood sample tube and stored at 4 °C for <6 h. DNA was extracted using the Blood Genome Column Medium Extraction Kit (Tiangen Biotech, Beijing, China) in accordance with the manufacturer’s instructions. Protein-coding exome enrichment was performed using the xGen Exome Research Panel v.1.0, which is composed of 429,826 individually synthesized and quality-controlled probes targeting 49.11 Mb of protein-coding regions (>23,000 genes) of the human genome. Whole-exon sequencing was performed using the NovaSeq 6000 platform (Illumina, San Diego, CA, USA), and the raw data were processed using FastP to remove adapters and filter out low-quality reads. Paired-end reads were aligned with the Ensembl GRCh38/hg38 reference genome using the Burrows–Wheeler Aligner tool. Variant annotation was performed in accordance with database-sourced minor allele frequencies (MAFs) and practical guidelines on pathogenicity that were issued by the American College of Medical Genetics. The annotation of MAFs was performed based on the 1000 Genomes, dbSNP, ESP, ExAC, Provean, Sift, Polypen2_hdiv, Polypen2_hvar, and Chigene in-house MAF databases using the R software program (R Foundation for Statistical Computing, Vienna, Austria). A novel mutation in *KCNH2* was finally identified. While the parents also received WES analysis, and both of them were absent of *KCNH2* c.1868C>A variant. The family pedigree tree of this proband had been presented in (Figure 3A). To our knowledge, *KCNH2* c.21868C>A has never before been reported in the 1000G and ExAC databases (Figure 3B). There was no other cardiomyopathy or arrhythmia-related genetic mutation identified by our whole-exon sequencing analysis. Besides, all the genetic variants related with sudden cardiac death (SCD) had been ruled out. As the parents of this neonate did not present with any symptoms of cardiovascular disease, the neonate was considered to be the proband.

MutationTaster and Polyphen-2 were used with the R software to predict the pathogenicity of *KCNH2* c.1868C>A and assess the effects of this mutation on the protein structure (Figure 3B). As there were *KCNH2* protein crystal structure data available, analysis was performed using SWISS-MODEL (https://swissmodel.expasy.org/) with the 5k7l.1.A template (Figures 3E,G). The capability of the protein structure was estimated using Ramachandran plots (Figures 3F,H). The differences between wild-type and *KCNH2* p.T623N mutant crystal structures were estimated by ensemble variance (Figures 3C,D). Changes in the free energy of the model were estimated using the mutation cutoff scanning matrix (mCSM) method (http://biosig.unimelb.edu.au/mcsm/). The signature vector that was ultimately generated was used to train the predictive classification and regression model for calculating the change in the Gibbs folding free energy induced by the mutations.

Based on laboratory analyses and the neonate’s clinical manifestations, a genetic disorder was strongly suspected. Whole-exon sequencing was performed using the Illumina NovaSeq 6000 platform, and a *de novo* c.1868C>A (p.T623N) heterozygous mutation was identified in the *KCNH2* gene. Neither of the neonate’s parents carried this variant. According to the American College of Medical Genetics, these variants have an uncertain pathogenicity (PM1+PM2_Supporting + PP3). The variant we identified, *KCNH2* c.1868C>A, had not been reported in any population. This is the first report of this variant. An analysis performed with MutationTester revealed that this mutation is considered pathogenic due to amino acid sequence changes, the protein features affected, and a loss of helix superstructure (probability = 0.999 for c.1868C>A). PolyPhen 2.0 predicted this mutation of p.T623N to be “probably damaging” (score = 1.0, sensitivity = 0.00, and specificity = 1.00). The SWISS-MODEL tool was used to analyze stability after amino acid changes. Ramachandran plots indicated that amino acid positions were altered (Figures 3F,H). Rebuilding the molecular structure based on a 5k7l.1.A template resulted in the identification of residue changes at amino acid position 623 (Figures 3E,G). Using SWISS-MODEL protein stability prediction tools, ensemble changes among the encoded amino acids exhibited significant variance. Three types of calculation methods all demonstrated significant destabilizing changes (mCSM = −0.338 kcal/mol).

**FIGURE 4:**
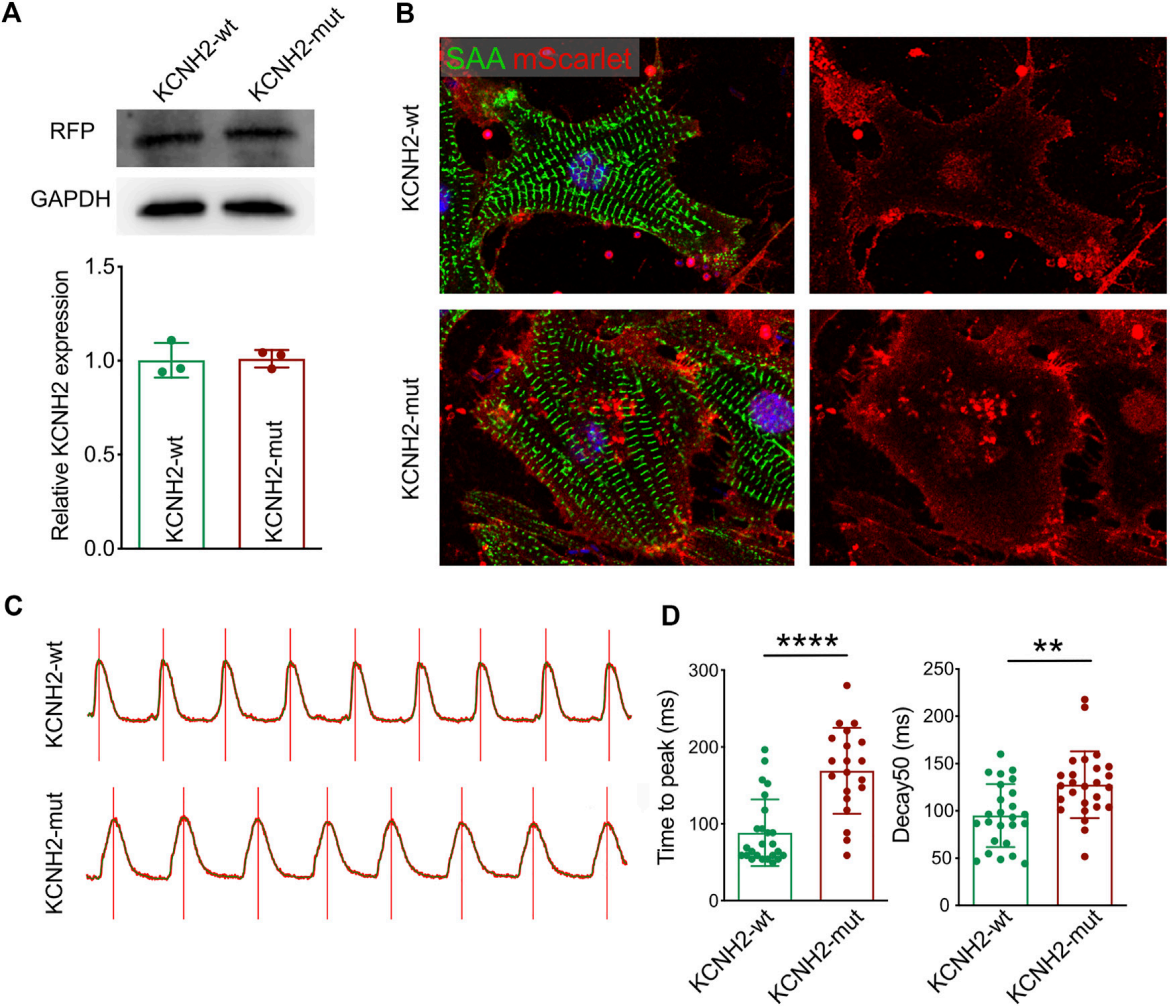
Functional validation of *KCNH2* c.1868C>A mutation on cardiomyocyte. **(A)** Western blotting demonstrated the similar expression of KCNH2 protein between wild type and mutation. **(B)** Immunofluorescence staining revealed the successful infection of KCNH2 overexpression plasmids in cardiomyocytes. **(C)** Calcium transit waves between KCNH2-wt and KCNH2-mut plasmids transfected cardiomyocytes. **(D)** The increased duration of time to peak and decay50 was identified in KCNH2-mut cardiomyocytes. RFP, red fluorescence protein; SAA, sarcomeric *α*-actinin. **, *p* < 0.01; ****, *p* < 0.0001.

To validate the functional impairment of *KCNH2* c.1868C>A, two kinds of overexpression plasmids were established as wild type human *KCNH2* (KCNH2-wt) and c.1868C>A allele *KCNH2* (KCNH2-mut) based on the pgl3 basic vector, and both KCNH2 molecules (wt and mut) were liganded with mScarlet by P2A (pgl3-KCNH2-wt-P2A-mScarlet and pgl3-KCNH2-mut-P2A-mScarlet). Then neonatal mice ventricular myocytes (NMVMs) were isolated using the Neomyt Kit (Cellutron, NC-6031) and plated on 1% Matrigel (Corning, 354234)-coated plates in cardiomyocyte culture media (low glucose DMEM (Gibco), 5% horse serum (American Type Culture Collection, 30–2040), and 2% chicken embryo extract (VWR, 100356-958)). The following day, two plasmids were transfected into NMVMs. Protein was extracted by the RIPA lysis buffer system (Santa Cruz Biotechnology, sc-24948) with Mini Protease Inhibitor Cocktail Tablets (cOmplete, 4693124001). The expression of KNCH2 was assessed by Western Blotting of mScarlet (Figure 4A). NMVMs were harvested on the 7th culture day and fixed using paraformaldehyde. Then, immunofluorescence was performed to demonstrate the expression of transfected KCNH2 (mScarlet), while cardiomyocytes were stained with SAA (Figure 4B). Intracellular Ca^2+^ recordings were performed after loading with Fluo-4 AM (10 μM, Invitrogen, F14217) for 30 min. After loading, NMVMs were subsequently washed with a normal Tyrode solution (140 mM NaCl; 4 mM KCl; 1 mM MgCl2; 1.8 mM CaCl2; 10 mM Glucose; and 5 mM HEPES, pH 7.4, adjusted with NaOH) to remove the excess dye for 20 min. All image data were acquired using an FV3000 confocal microscope (Figure 4C), which demonstrated the prolonged duration of pgl3-KCNH2-mut-P2A-mScarlet infected NMVMs both in time to peak and decay50 (Figure 4D). Thus, this experiment validated the impaired KCNH2 function-carrying c.1868C>A allele.

### Outcome and follow-up

After the neonate was transferred to the cardiac intensive care unit, a temporary pacemaker was implanted for the positive treatment. Once the genetic result of *KCNH2* c.1868C>A was identified, the neonate remained at an extremely high risk of SCD. Thus, a permanent pacemaker was a therapeutic alternative. The neonate’s parents agreed to the implantation of a permanent pacemaker, and thus, the patient planned to receive an epicardial pacemaker implantation between 3 and 4 months. Additionally, her parents declined to have any medication administration. During two months of postnatal follow-up, the patient did not suffer from syncope or heart dysfunction. However, the body length remained approximately -3.2 SD from the reference value following prenatal long-term DEX exposure, which revealed a developmental restriction. Unfortunately, the patient died from SCD around 2.5 months of age before the scheduled date of pacemaker implantation. Suspected fatal arrhythmia, such as torsades de pointes due to LQTS, was likely the main reason for the induction of death.

## Discussion

Inherit arrhythmias lead to higher risks of heart dysfunction, even SCD, in early childhood (Dai et al., 2021). However, most types of inherit arrhythmias have not been identified in fetuses, which indicates a low intrauterine penetrance. Generally, maternal autoimmune diseases impair the conduction system by transplacental transfusion of immune antibodies that attack the heart conduction system and result in localized fibrosis (Suryawanshi et al., 2020). Maternal autoimmune diseases and autoimmune antibodies, especially high-titer anti–SSB/La antibodies and particularly when accompanied by high-titer anti–SSA/Ro antibodies, may cause congenital AVB, sinoatrial node dysfunction, ventricular and junctional tachycardias, and long QT intervals (Wainwright et al., 2020). Indeed, autoimmune factors contribute to more than half of fetal bradycardia cases. However, a large proportion of fetal bradycardia cases lack any evidence of immune involvement. The DEX administration strategy for such cases is critical and challenging.

Congenital LQTS, which is an autoimmune antibody-negative fetal bradycardia, is a cause of fetal AVB (Otsuki et al., 2020). The occurrence of AVB in concert with LQTS is not due to disease of the conduction system itself but rather due to the prolonged ventricular repolarization time that leads to atrial activation before ventricular repolarization is complete (Batra and Balaji, 2019). Generally, QT interval prolongation is responsible for bradycardia (Oka et al., 2010). In a report from Garson et al. that included a series of 287 patients with LQTS, there were 15 patients (5%) with AVB, 13 patients with 2:1 AVB, and only 2 (0.7%) patients with congenital AVB (Mendoza et al., 2010). Early awareness of LQTS is critical in preventing adverse clinical outcomes. Once AVB induced by QT prolongation occurs, it carries a high risk of TDP, which is closely associated with SCD (Rosso et al., 2014). Intrauterine LQTS-induced 2:1 transduction AVB is associated with higher mortality due to fetal hydrops and cardiac dysfunction. In such cases, postnatal genetic screening is recommended by the American Heart Association (AHA) to reach a definitive diagnosis as most conditions of congenital LQTS do not require any treatment during infancy; treatment is only recommended at the onset of ventricular tachycardia or TDP.

According to most protocols or guidelines of fetal arrhythmia management, LQTS should be highly suspected in fetal bradycardia cases, especially for the 2:1 transduction AVB (Kasar et al., 2019), and postnatal genetic screening is recommended to reach a definitive diagnosis. However, if there is no positive family history, it may be difficult to distinguish LQTS from other types of fetal bradycardia using fetal ECG. AHA guidelines recommend fetal magnetocardiography or ECG to monitor the QTc interval; however, there is still a gap in illustrating the actual QTc interval. Fetal therapy with DEX is strongly recommended for autoimmune-mediated fetal bradycardia. DEX treatment of fetuses with established CHB and without heart failure might also be considered with the goal of improving survival or reducing the incidence of dilated cardiomyopathy. The usefulness of DEX therapy in this population has not been established given that current studies have been retrospective and nonrandomized and have had an incomplete follow-up. Generally, DEX is also provided to autoimmune antibody-negative fetuses with DEX dosage reduction based on whether the fetal CHB may be terminated or unchanged after four weeks of treatment. Moreover, if the high-degree fetal AVB changed to first-degree AVB or non-threatening fetal bradycardia, a small dosage of DEX is always continuously administrated to birth. Recently, the long-term adverse effects of prenatal DEX exposure have been noted by researchers (Pal et al., 2022; Shinwell et al., 2022; Wang et al., 2022). In experimental animals, pulmonary fibrosis, osteoporosis, long bone growth impairment, and neonatal hypoglycemia were identified following prenatal DEX exposure (Rivet et al., 2022; Wu et al., 2022; Xie et al., 2022; Zhang et al., 2022). Mechanistically, circadian rhythms, insulin-like growth factor 1 signaling, angiotensin II signaling, histone modifications, and dysregulation of miRNAs were responsible for long-term adverse presentations following DEX exposure (Han et al., 2022; Liu et al., 2022; Madhavpeddi et al., 2022; Murray et al., 2022; Qiu et al., 2022). Thus, the benefits from prenatal DEX administration for autoimmune antibody-negative patients are still being debated.

LQTS is a type of inherit cardiovascular disease that induces the prolonged duration of QT interval. Besides, some cases also demonstrated that LQTS presents as AVB (Aziz et al., 2010; Oka et al., 2010; Sun et al., 2022). It has been considered that AVB could be due to LQTS as a kind of pseudo AVB. The variant of KCNH2 would lead to the loss-of-function of this molecule. The delay repolarization would cause the next beating signaling from atrioventricular node to be irresponsive, which would induce the 2:1 AVB. In this case, the fetus presented with 2:1 transduction AVB at the first fetal ECG, and no positive results had been identified for autoimmune antibodies or potential maternal-related disease screenings. However, the patient initially received DEX therapy, and the high-degree AVB regressed into a mild prolonged AV interval, indicating some positive effects of the DEX treatment. Thus, continuous small dose DEX administration was provided until delivery. After birth, LQTS was confirmed by ECG and genetic screening. Unfortunately, the neonate developed growth restriction postnatally. Subsequently, the case was brought to us to review the treatment strategy for prenatal CHB without positive evidence of autoimmune antibodies and for a critical discussion of the benefits of DEX exposure. Importantly, prenatal genetic screening should be highly recommended for autoimmune antibody-negative fetuses to exclude any genetic syndromes and channelopathies. With the rapid development of next generation sequencing technologies, the expense and duration of such methods have been significantly reduced and allow timely and convincing molecular results. Additionally, the shifted diagnostic timepoint of LQTS or other types of channelopathies may help to avoid unnecessary DEX prenatal exposure.

## Conclusion

Prenatal management of fetal CHB with DEX benefits neonates with high-degree AVB and positive autoimmune antibody tests. However, genetic screening should be recommended for fetuses with autoimmune antibody-negative high-degree AVB, especially those with 2:1 AVB and in those cases in which changes in fetal heart rhythm occur with initial DEX treatment. Genetic screening may help identify genetic variant–related channelopathies and is critical for avoiding unexpected prenatal DEX exposure and its long-term adverse postnatal complications.

## Data Availability

The original contributions presented in the study are included in the article/Supplementary Material; further inquiries can be directed to the corresponding authors.

## Abbreviations

ANAs, anti-nuclear antibodies; AVB, atrioventricular block; CAVB, complete atrioventricular block; DEX, dexamethasone; ECG, electrocardiogram; LQTS, long QT syndrome; MAFs, minor allele frequencies; SCD, sudden cardiac death; SDM, site-directed mutator; TDP, torsades de pointes.
