# Supplementary figures and images for: Case Report: A novel KNCH2 variant-induced fetal heart block and the advantages of fetal genomic sequencing in prenatal long-term dexamethasone exposure
Source: Front Genet. 2022 Nov 29;13:1010078. doi: 10.3389/fgene.2022.1010078 (PMC9745090; doi:10.3389/fgene.2022.1010078)

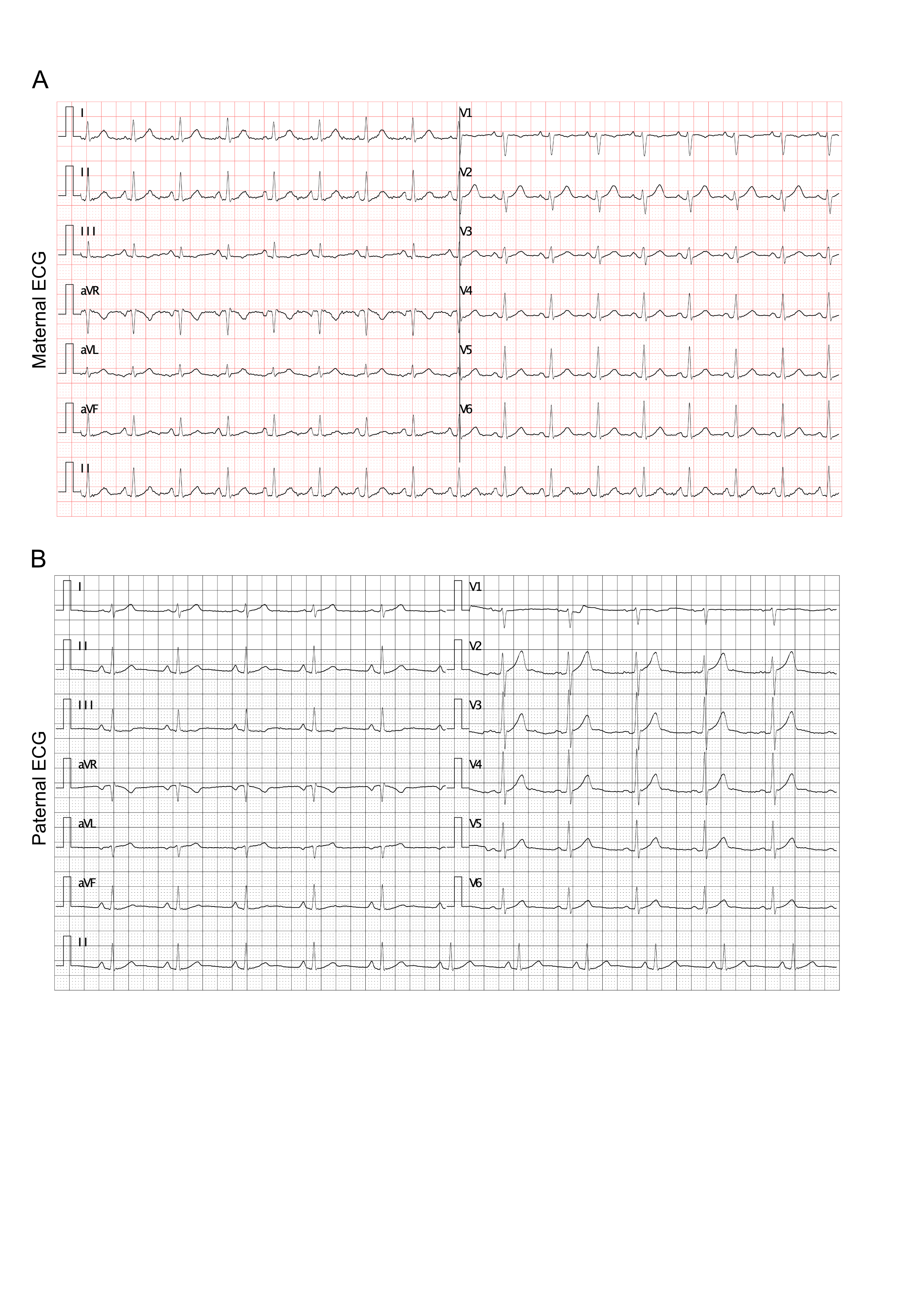

Supplement: Supplementary file 1 [file Image1.JPEG]
